# Editorial: Combating antimicrobial resistance: peptides and other novel therapeutic interventions to treat ocular, oral and skin infections

**DOI:** 10.3389/fcimb.2024.1388744

**Published:** 2024-03-14

**Authors:** Sanhita Roy, Peter N. Monk

**Affiliations:** ^1^ Prof. Brien Holden Eye Research Centre, LV Prasad Eye Institute, Hyderabad, India; ^2^ Dr. Chigurupati Nageswara Rao Ocular Pharmacology Research Centre, LV Prasad Eye Institute, Hyderabad, India; ^3^ Department of Infection, Immunity and Cardiovascular Disease, University of Sheffield, Sheffield, United Kingdom

**Keywords:** antimicrobial resistance, antimicrobial peptides, biofilm, small molecules, antibiotics

## Introduction

The discovery of antibiotics and antimicrobials are arguably the most important addition to the powerful arsenal of weapons against human disease. However, the rapid emergence of antimicrobial resistance (AMR) against existing drugs is becoming a global health challenge. World Health Organization predicts almost 10 million deaths per year from AMR by 2050 (Antimicrobial Resistance, 2022), which will cause a huge socio-economic burden. AMR increases patient morbidity, mortality and healthcare costs significantly. AMR results from increased adaptation of the microbes over time towards the existing antimicrobials, which is often achieved by horizontal transfer of antibiotic resistance genes (Walsh, 2000). Due to non-judicious use of antimicrobials over the last few decades, there has been evolution and spread of resistant genes among pathogens causing emergence of multi-drug resistant and extremely drug resistant pathogens. The ability to form biofilm is one of the most common mode by which pathogen exhibits AMR and often the mechanisms are complex (Stewart and Costerton, 2001). The AMR in biofilm is typically regulated by different factors. One explanation is the limited penetration of the drug in the biofilm network thus preventing the attainment of effective concentration, also there are increased chances of sequestration of the antibiotic by extracellular matrix environment (Stewart, 1996). The altered environment within the biofilm with different pH and anaerobic niches are also responsible for increased resistance to the biofilms (Urwin et al., 2020). The biofilm specific resistance is also due to existence of ‘persister’ cells that constitutes a small proportion of the total population that evade the bactericidal effects of antibiotics by downregulation of genes involved in metabolic pathways (Fisher et al., 2017). Another major consequence of AMR is that it causes delayed wound healing, mostly due to presence of biofilms that occur in 60-100% of chronic wounds (Anderl et al., 2000).

## Potential solutions

In depth research is being carried out to develop new antimicrobial strategies in order to prevent increase in AMR. One such strategy is to use different small molecule virulence inhibitors that prevent infection but do not kill the bacteria directly (Sharma et al., 2020). In a similar way, novel anti-adhesion peptides have been designed that mainly target the adhesion of the bacteria to the host cells, which is usually the first step to initiate infection. One such approach is tetraspanin derived peptides that have shown potential in inhibiting infection both *in vitro* and *in vivo* and also accelerated wound healing in murine corneas (Jadi et al., 2023). Along with these, novel therapeutic interventions in form of antimicrobial peptides can be used to prevent AMR. The repurposed drugs which are already approved and have low safety risks are often preferred due to reduced development timelines and cost compared to *de novo* discovery of new agents can be explored to combat AMR (Farha and Brown, 2019).

In this Research Topic, we put forward a collection of original research articles that studied different antimicrobials in combating infection and biofilm formation using *in vitro* and *in vivo* models of infection.

## Redesign of existing antimicrobials

The study by Thamilselvan et al. highlights designing of new molecules from the existing repertoire of lead compounds by adding small molecules replacing the boronic acid moiety. They have utilized several interdisciplinary aspects of bioinformatics, medicinal chemistry and microbiology. The several efflux pumps of *S. aureus* strains including norA play important roles in the emerging resistance against different antibiotics. The authors modelled the 3D structure of NorA and 5-NPPP was selected out of 42 compounds based on the docking score. They checked the combinatorial effect of ciprofloxacin and 5-NPPP against clinical isolate of *S. aureus* and found that ciprofloxacin showed strong additive effect against 5-NPPP. The compound further decreased NorA mediated efflux in bacteria and was not cytotoxic to mammalian cells even at 100X MIC.

## Low toxicity antimicrobial

Somayajulu et al. has studied the effect of pH of glycyrrhizin (GLY) on normal and wounded cornea and conjunctiva using mice model. The authors have earlier shown the effectiveness GLY, a derivative of licorice root, against *Pseudomonas aeruginosa* keratitis using both clinical and laboratory strains. In this article they have demonstrated no alteration in assembly of subbasal nerve fibres or nerve density on application of acidic or neutral GLY compared to PBS. Additionally, no difference in wound closure was observed 24 h post wounding was observed in acidic or neutral GLY treated corneas compared to PBS, however neutral GLY fared over acidic GLY in reducing the bacterial count in mice model of corneal infection.

## Adjuvants to improve antimicrobial activity

In the current article Rasha et al. used a zinc oxide nanoparticle (ZnO-NP) to inhibit carbapenem resistant *Klebsiella pneumoniae* both *in vitro* and *in vivo*. These nano particles were prepared biogenically using *Aspergillus niger* and were characterized using infrared spectroscopy. These nanoparticles caused damage of bacterial membrane and inhibited the growth of *K. pneumoniae*. The authors also demonstrated that these particle causes significantly improved wound healing compared to imipenem treated wounds in rats.

## Overcoming biofilms

Biofilms are often associated with increased resistance of the pathogens towards antimicrobials and is associated with serious infections. In the presence of vast array of antimicrobial peptides (AMPs), often it becomes very challenging and time and resource intensive to identify the potential AMP that act against biofilms. Mhade et al. have manually curated a repository of AMPs focusing on the structural and functional aspect for peptides effective against biofilms. This also includes information on the source, experimental testing and potency of the peptides with feasible in silico evaluation. They also demonstrated a case study against *Corynebacterium striatum*, a multidrug resistant biofilm forming bacteria. A selected AMP was evaluated by *in silico*, and *in vitro t*esting. Homology modelling was also performed between the candidate AMP and *C. striatum* protein.

## Conclusion

In conclusion, new strategies need to be implemented to prevent AMR including determination of novel compounds, repurposing of existing drugs, natural product based agents, and combinatorial therapies between peptides and existing antibiotics. Moreover, special care and attention must be implemented regarding the biosafety and biocompatibility while designing new antimicrobials for *in vivo* applications. In-depth studies must be done to explore alternative therapeutic intervention and efficient drug delivery method to disrupt biofilms and battel antimicrobial resistance.

## Author contributions

SR: Writing – original draft, Writing – review & editing. PM: Writing – original draft, Writing – review & editing.
